# Ultrasound Guided Intravenous Access by Nursing versus Resident Staff in a Community Based Teaching Hospital: A “Noninferiority” Trial

**DOI:** 10.1155/2015/563139

**Published:** 2015-08-30

**Authors:** Thomas Carter, Chris Conrad, J. Link Wilson, Godwin Dogbey

**Affiliations:** ^1^Emergency Department, Southern Ohio Medical Center, Portsmouth, OH 45662, USA; ^2^CORE Research Office, Ohio University Heritage College of Osteopathic Medicine, Athens, OH 45701, USA

## Abstract

*Objectives*. Ultrasound (US) guidance is a safe and effective method for peripheral intravenous (IV) catheter placement. However, no studies have directly compared the success rate of emergency medicine (EM) residents and nurses at using this technique especially in community hospital settings. This prospective “noninferiority” study sought to demonstrate that nursing staff are at least as successful as EM residents at placing US guided IVs. *Methods*. A group of 5 EM residents and 11 nurse volunteers with at least two years' experience underwent training sessions in hands-on practice and didactic instruction with prospective follow-up. Two failed attempts on a patient using standard approach by an emergency department (ED) nurse were deemed to be “difficult sticks” and randomly assigned to either a nurse or resident, based on the day they presented. *Results*. A total of 90 attempts, consisting of trials on 90 patients, were recorded with a success rate of 85% and 86% for residents and nurses, respectively. With a *p* value of .305, there was no statistically significant difference in the success rate between the residents and nurses. *Conclusion*. Properly trained nursing staff can be as equally successful as EM residents in placing US guided intravenous lines.

## 1. Introduction

The use of ultrasound (US) technology in placing peripheral lines has been shown to reduce the need for central venous catheters—a more painful process with more grave possible complications [1, 2]. Nurses are at the frontline of clinical contact with patients in the emergency department (ED). As a result, there have been repeated calls for training of nurses on the use of US guided IV (USGIV) access in the ED and hospital setting, in general [3]. In response to such calls, programs for nurses have been designed and implemented [4, 5]. When nursing staff in the ED of a hospital are unable to obtain peripheral venous access after multiple attempts, they have the option to forego any venous access, request to place a central venous catheter, or request ultrasound (US) guided IV access by a physician [2, 6]. This is particularly the case in most community teaching hospitals.

Multiple studies [7–10] have demonstrated the efficacy of nurses and ED technicians using US for IV access. This finding has not been widely used and in some institutions a culture of “hands off” for nurses and technicians still exists around ultrasound guidance for some procedures. The emergent issue, therefore, has not been over whether ultrasound is safe and effective but about who should be able to use the machines and for what purpose.

In underserved community hospitals, such as the one under study, low levels of workforce have necessitated that limited available human resources are utilized to the fullest in patient care. Consequently, nurses and residents face the task of performing many procedures including IV placements frequently. Anecdotally, it was observed that fear has led to low levels of comfort among nurses regarding the use of US as a guide for IV access and placement in patients especially those with difficult access, “difficult sticks.” The training requirements of emergency residents and physicians have meant that, to a large extent, they are relatively facile with USGIV access and placement [6]. In light of this, it is important within a community hospital environment to have nurses at par with residents with regard to success in placing USGIVs. However, there are no studies found in the literature comparing the efficacy of ultrasound use by nurses and residents in situations where US guided access to IVs is necessary for the patient.

From the foregoing, therefore, the goal of our study was to directly compare success rate between emergency medicine (EM) residents and emergency room nursing staff at obtaining peripheral venous access under real time ultrasound guidance. We assert that the nursing staff can be, if properly trained, as effective as the emergency medicine residents at this technique.

## 2. Methods

### 2.1. Study Design and Setting

This single center, nonblinded, quasi-randomized, “noninferiority” study was conducted in a 180-bed Midwestern community teaching hospital in the United States. Local Institutional/Ethics Review Board approval was obtained for this study.

### 2.2. Population and Sample

**Figure 1:**
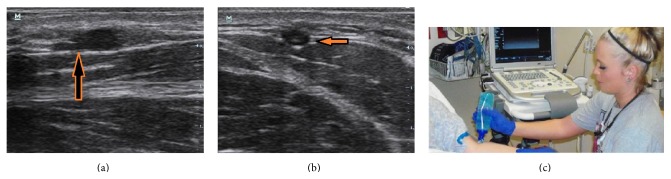
(a) Catheter tip (hyperechoic or white) shown accessing a vein (hypoechoic or black square) in the oblique access; the catheter is highlighted in by the orange arrow. (b) A catheter tip (hyperechoic or white) shown accessing the peripheral vein (hypoechoic or black circle) circle in the coronal plane delineated by the orange arrow. (c) A nurse preparing to perform US guided IV access notes the location of the ultrasound machine to allow for line of site of both the machine and the patient.

A sample of nurses and residents was self-selected from the population of nurses and emergency medicine (EM) residents in the community teaching hospital. The nursing staff in the emergency department of the community teaching hospital who consented to participate in the study was trained on use of the ultrasound machine through hands-on instruction followed by simulated venous canalization in model gel (see Figures 1(a), 1(b), and 1(c)). After the nurses were introduced to the technique, we determined the extent to which they could effectively use US guidance in the appropriate situations, as directed by a physician, to obtain IV access in situations when it was difficult to access patients.

We began the trial with the self-selected group of 11 highly motivated nurse volunteers who had successfully completed the training sessions. Since nurses in the ED were already quite experienced in IV access, the learning curve was short and only related to visualization on the US screen and the dexterity to insert IV while holding the probe and watching the screen (Figures 1(a) and 1(b)). Setup with the screen at the patients head to allow proper visualization and to maintain orientation with the field was recommended as displayed in Figure 1(c). The group of nurses was pitted against a group of five (5) equally trained and motivated emergency medicine residents who had received one or more years of on-the-job training in ultrasound use for various procedures, including peripheral venous access.

### 2.3. Inclusion and Exclusion Criteria

Inclusion criteria comprised adult patients (18 years or older) who needed peripheral venous access as judged by the treating attending physician. Furthermore, patients should have undergone two unsuccessful attempts at peripheral venous access by anatomic landmarks by any ED nurse, as is the standard procedure at this time. Those excluded from the study were pediatric patients (less than 18 years old), patients with an urgent need for central venous access due to critical status, hemodialysis patients, mastectomy or radiation therapy patients, patients randomly placed in a room to which a participating nurse was not previously assigned, and/or patients who had successful peripheral venous access in two or less attempts using the traditional anatomic landmarks technique. Ultrasound guided anatomy of the upper extremity was the only decision for access location; no standardized vein was required and only intermediate depth vessels were utilized.

### 2.4. Study Protocol and Subject Assignments

Quasi-randomization was done with patients assigned to rooms based on presentation before peripheral access was ordered. One or more nurses covered the patient room before patient assignment or any order for peripheral venous access was identified. The study nurse assigned to a room continued care after patient consent was obtained and attempts to perform the US guided IV when a nonstudy nurse was unsuccessful using the traditional techniques. Similarly, after patient consent, a resident attempted to perform the ultrasound guided placement. A comparison of the success rates in obtaining a working IV between the nurse and resident groups was made. Success (hit) was defined as the withdrawal of blood from the catheter followed by the ability to freely flush saline without any signs of infiltration.

### 2.5. Measurements

Data was collected simply by measuring either success or lack of success in canalization as defined above. The numbers were compared directly to answer our simple question of whether nurses can be equally efficacious in obtaining peripheral venous access with ultrasound guidance when compared to emergency medicine residents. The chi-squared test of association was used to determine overall relationship between the health professional type (nurses versus residents) and success at the IV placement as well as complications. Furthermore, the nonparametric chi-square test of equality of proportions was used to determine if success rates were different between the two groups. Statistical significance was set at *p* < .05. Data was analyzed using the SPSS version 21 (IBM, Chicago, IL).

## 3. Results

The inclusion criteria were met by 90 patients who consented to participate in the study over the period of interest. The 11 ED nursing staff made 50 attempts while the five EM residents made 40 IV placement attempts. As previously stated, the outcome measures of interest were the number of successes and complications associated with the ultrasound guided IV placement by each member of each of the two groups. There was no statistically significant association between the provider type and success at the IV placement, *p* = .893 (see Table 1).

In all, there were 77 hits out of the 90 attempts yielding an overall success rate of 85.5%. Nurses recorded 43 successes out of the 50 attempts representing a group success rate of 86% while residents scored 34 hits out of 40, a group success rate of 85%. The success rates between nurses and residents were not significantly different statistically, *p* = .305. Regarding complications, similarly no statistically significant relationship was found with provider type, *p* = .110. There were two out of the 90 attempts, a complication rate of 2.2% overall (see Table 2). These two resulted solely from the residents' attempts, a 5% group complication rate.

## 4. Discussion

The success rates of 85% and 86% observed for residents and nurses, respectively, in USGIV placements were consistent with results reported in some studies involving nurses [2, 7]. Those studies reported success rates in the range 53%–85% depending on technique (one-person versus two-person), type of vein (basilic versus brachial), number of attempts (one or multiple), and overall cannulation [11].

Our study observed no statistically significant difference in the success rate between the providers: nurses and EM residents. However, there was a measured difference in complications rate (5% for residents and none for nurses). Further studies involving larger sample size are needed to determine if that result was purely due to chance or a real difference. In this study, no overall association was found between complication and provider type (nurse or resident). Each provider did not work on the same patient more than once and so interoperator reliability (measured by* kappa*) was not relevant and not computed. Moreover, the primary outcome of success as defined was not based on interpreting the sonographic images thereby rendering the issue of reproducibility of images untenable. We did not determine time to success mostly due to the lower acuity of the patient with IV access needs.

## 5. Conclusion

Nurses have used ultrasound to perform peripheral IV insertion in the past but this study has provided further evidence that they are equally adept at this procedure as emergency medicine residents. This was in light of the fact that no significant difference was observed between residents and nurses with regard to the success rate of US guided IV placement. Based on this result, a protocol can be developed for training and routine use of ultrasound for peripheral IV insertion in the ED for nurses. Implementing a program for use of intermediate lines under ultrasound guidance for nurses could expedite care, reduce pain, and decrease possible complications associated with central access.

## Figures and Tables

**Table 1:** Relationship between provider type and success (hit) at ultrasound guided IV access.

Provider type	Hit		Total	*p* value
	Yes	No		
Resident	34	6	**40**	.893
Nurse	43	7	**50**	
Total	**77**	**13**	**90**	

**Table 2:** Relationship between provider type and complications in the use of ultrasound guided IV access.

Provider type	Complications		Total	*p* value
	Yes	No		
Resident	2	38	**40**	.110
Nurse	0	50	**50**	
Total	**2**	**88**	**90**	
